# Using Selective Agar Containing Ciprofloxacin and Tetracycline Reveals Resistant Oral Microbiota in Healthy and Periodontitis Patients

**DOI:** 10.1002/mbo3.70298

**Published:** 2026-04-23

**Authors:** Marietta Wolf, Jennifer Metz, Annette Wittmer, Klaus Pelz, Kirstin Vach, Christiane von Ohle, Diana Wolff, Cornelia Frese, Fabian Cieplik, Ali Al‐Ahmad

**Affiliations:** ^1^ Department of Operative Dentistry and Periodontology, Center of Dental Medicine, Medical Center & Medical Faculty University of Freiburg Freiburg Germany; ^2^ Private practice Kandel Germany; ^3^ Institute of Medical Microbiology and Hygiene, Medical Center & Medical Faculty University of Freiburg Freiburg Germany; ^4^ Institute of Medical Biometry and Statistics, Faculty of Medicine University of Freiburg Freiburg Germany; ^5^ Centre for Data Science Eberswalde University for Sustainable Development Eberswalde Germany; ^6^ Department of Conservative Dentistry and Periodontology University Hospital Tübingen Tübingen Germany; ^7^ Department of Conservative Dentistry, Clinic for Oral, Dental and Maxillofacial Diseases University Hospital Heidelberg, Heidelberg University Heidelberg Germany

**Keywords:** ampicillin, antibiotic resistance, antimicrobial resistance, ciprofloxacin, periodontitis, tetracycline

## Abstract

The oral cavity may act as a reservoir for antibiotic resistance. This study aimed to directly isolate and identify phenotypically resistant bacteria from the oral biofilm of healthy individuals and patients with periodontitis, using tetracycline, and ciprofloxacin containing selective agar. Furthermore, resistance of selected bacteria towards ampicillin was also evaluated. Plaque samples were collected from 12 patients (six healthy, six with periodontitis). Bacteria were cultured on selective agar containg defined antibiotic concentration and non‐selective media under aerobic and anaerobic conditions, identified by MALDI‐TOF mass spectrometry and 16S rDNA sequencing. The selected bacteria were subsequently tested for susceptibility using disk diffusion, E‐test, and β‐lactamase assay. 495 strains representing 106 species were isolated, including 54 aerobes/facultative anaerobes and 52 obligate anaerobes. Antibiotic resistance was observed in all subjects: 15.2% of isolates were resistant to tetracycline, 32.9% to ciprofloxacin, and 0.6% to ampicillin, with no significant differences between healthy and periodontitis groups. Tetracycline resistance was most frequent in the *Streptococcus mitis* group and *Eubacterium* spp., while ciprofloxacin resistance was dominated by *Actinomyces‐Schaalia* group. Concluding, prevalence of antibiotic‐resistance was comparable between healthy and periodontitis patients. Resistance was most prevalent against ciprofloxacin and tetracycline, highlighting the oral cavity as a relevant reservoir for antibiotic resistance.

## Graphical abstract

Oral biofilms from healthy and periodontitis patients harbor diverse bacteria, including aerobes and obligate anaerobes. Using selective media with tetracycline or ciprofloxacin, 495 strains (106 species) were isolated. Resistance was common—32.9% to ciprofloxacin, 15.2% to tetracycline, 0.6% to ampicillin—highlighting the oral cavity as a reservoir for antibiotic resistance.

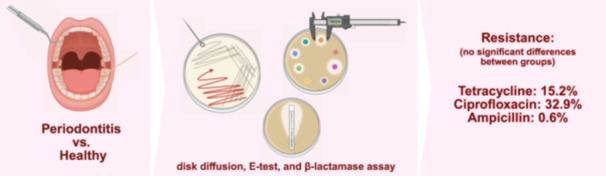

## 1 Introduction

The human oral cavity comprises a variety of surfaces, including the tongue, mucosa, gingiva, palate, teeth and gingival sulcus, which together support a complex microbial ecosystem. Oral bacteria form structured biofilms in which they can communicate via quorum sensing, share nutrients (Wright et al. 2013; Jakubovics et al. 2021), and exhibit up to 1,000 times higher tolerance to antibiotics than their planktonic counterparts (Olsen 2015). The widespread use of antibiotics, alongside natural selective pressures, has led to the emergence of resistance against different antibiotics among bacteria. Resistance can be inherited through point mutations or transferred horizontally via plasmids and other mobile genetic elements. Horizontal gene transfer, particularly transformation and conjugation, is significantly enhanced within biofilms and plays a major role in spreading resistance genes (Roberts and Mullany 2010). Metagenome studies also show that many antibiotic resistance genes are located on mobile genetic elements, which further facilitates horizontal gene transfer (Sukumar et al. 2016; Anderson et al. 2023; Bartsch et al. 2024).

An imbalance between the host and its oral microbiota can result in dysbiosis and diseases such as periodontitis, which is a chronic inflammatory condition affecting the periodontium (Lasserre et al. 2018; Kinane et al. 2017). Periodontitis has multiple risk factors, including microbial, immunological, genetic and environmental factors (Dentino et al. 2013). Antibiotics are often used in periodontal therapy, either systemically or locally. While systemic antibiotics can improve treatment outcomes, they may also lead to adverse effects and promote resistance (Jepsen and Jepsen 2016). Local antimicrobials deliver high concentrations to the infection site while posing fewer systemic risks (Jepsen and Jepsen 2016) but also less effects and are typically used for single deep persistent pockets (American Academy of Periodontology Statement on Local Delivery of Sustained or Controlled Release Antimicrobials as Adjunctive Therapy in the Treatment of Periodontitis 2006).

In 2019 alone, antimicrobial resistance (AMR) was linked to 4.95 million deaths worldwide, with 1.27 million directly attributable to bacterial AMR (Murray et al. 2022). Projections suggest that by 2050, AMR‐related mortality will exceed cancer, claiming over 10 million lives annually (Naghavi et al. 2024; Thompson et al. 2025). Dentists contribute approximately 10% of all antimicrobial prescriptions globally (varying by country) (World Dental Federation 2020), underscoring their critical role in limiting AMR through judicious prescribing. Alarmingly, substantial antibiotic use is administered without adherence to clinical guidelines (Cope et al. 2016; Durkin et al. 2018). Interestingly, the oral microbiome offers a broad spectrum of AMR genes, many of which are located on mobile genetic elements capable of promoting horizontal gene transfer (Brooks et al. 2022a).

In this study, the impact of tetracycline, ciprofloxacin, and ampicillin on oral microbial communities was investigated. Tetracyclines, commonly applied topically in dentistry, have led to the emergence of diverse resistance mechanisms and a wide range of resistance genes within the oral microbiota (Villedieu et al. 2003; Lancaster et al. 2005; Roberts and Mullany 2011; Reynolds et al. 2016). Ciprofloxacin, a broad‐spectrum fluoroquinolone primarily used for systemic infections (Heidelbaugh and Holmstrom 2013; Lode 2014), has only rarely been associated with resistant oral isolates, although cases have been documented (Wade 1989; Rashid et al. 2015; Ehrmann et al. 2016, 2017). Ampicillin, in clinical use since 1961, targets both Gram‐positive and Gram‐negative bacteria and is used in dental prophylaxis and therapy for periodontitis and abscesses. Unsurprisingly, several ampicillin‐resistant oral bacteria have also been identified (Van Winkelhoff et al. 1997; Fosse et al. 2002; Gaetti‐Jardim et al. 2010; Rams et al. 2013).

While antibiotics such as clindamycin, amoxicillin‐clavulanate, and metronidazole are frequently recommended in dental practice, this study deliberately focused on tetracycline, ciprofloxacin, and ampicillin to assess under‐explored phenotypic resistance patterns across distinct oral microbiota in healthy individuals and periodontitis patients. Using culture‐based methods and susceptibility testing, this work aimed to assess phenotypic resistance profiles and explores their relationship with periodontal health status.

## 2 Material and Methods

### 2.1 Patient Selection

This study was approved by the Ethics Committee of Albert‐Ludwigs‐Universiy, Freiburg, Germany, with the study number 604/16, the Ethics Committee of Heidelberg Medical Faculty, Heidelberg, Germany, with the study number S‐652/2016, as well as by the Ethics Committee of Tuebingen University Hospital, Tuebingen, Germany, with the study number 863/201BO2. The research protocol complied with the principles of the Declaration of Helsinki. Samples of twelve patients who attended the centers of dental medicine of the University Medical Centers Freiburg and Heidelberg were obtained between April 2017 and March 2018. Inclusion criteria for all patients were age of at least 18 years, no pregnancy or lactation, no physical limitations affecting oral hygiene performance, no antibiotic intake within the last 6 months, no professional dental cleaning in the past 6 months, and no oral hygiene procedures (toothbrushing, mouthrinse) within 24 h prior to sample collection. Furthermore, healthy patients had to provide a DMFT (decayed, missing, filled teeth) score of 0, periodontal health (PSI grade 0–2), a generally healthy medical status, no diagnosed diabetes, no known severe systemic diseases (e.g., HIV, immunosuppression), no medications known to affect periodontal health (such as antiepileptics or antihypertensives), no pathological changes of the oral mucosa, and no interdental hygiene procedures within 72 h. The periodontitis group comprised individuals with Stage II to Stage IV localized or generalized periodontitis. To avoid disruption of the subgingival biofilm and transient influence on the resistome, subjects were excluded if they had received periodontal therapy within the last 24 months, or antibiotics or subgingival debridement within the last 6 months (Rashid et al. 2015; Paster et al. 2001).

### 2.2 Sample Collection, Isolation and Identification of Bacteria

**Figure 1:**
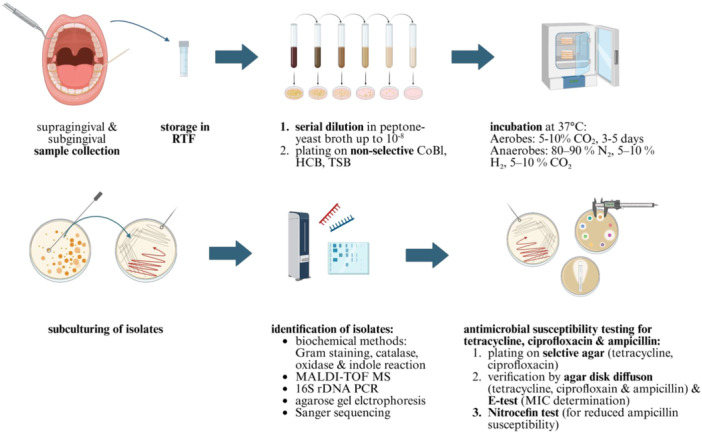
Workflow of study design and methodology. Samples were collected according to strict inclusion criteria. Supragingival (healthy group) or subgingival (periodontitis group) plaque samples were obtained, stored in reduced transport fluid at −80°C, serially diluted, and plated on selective and non‐selective agar media under aerobic and anaerobic conditions. Pure isolates were identified by morphological and biochemical methods, MALDI‐TOF MS, and 16S rDNA sequencing. Antimicrobial susceptibility testing was performed for tetracycline, ciprofloxacin, and ampicillin using selective agar plates, disk diffusion, E‐test, and β‐lactamase detection where appropriate.

In the healthy control group, supragingival plaque samples were obtained from all four quadrants using sterile Gracey curettes. For the periodontitis group, subgingival plaque was recovered using two or three sterile paper points per tooth, placed in the sulcus for 20 s (in total six paper points per patient). Samples were stored in sterile cryogenic tubes (Nunc, Thermo Fisher Scientific, Waltham, MA, USA) pre‐filled with 1.5 mL of reduced transport fluid (RTF) and stored at −80°C until further processing. Serial dilutions were prepared in peptone‐yeast broth up to 10^–8^, and aliquots from each dilution were plated on Columbia blood agar (CoBl), yeast‐cysteine blood agar (HCB), TSBV agar, and on selective CoBl and HCB plates containing tetracycline or ciprofloxacin. Selective agar (16 µg/mL tetracycline, 4 µg/mL ciprofloxacin) facilitated the isolation of low‐abundance species, with resistance interpreted according to EUCAST breakpoints. No selective agar for ampicillin was used due to its instability in growth media. Aerobic organisms were incubated at 37°C in 5%–10% CO₂ for three to 5 days. Anaerobes were incubated anaerobically for up to 8 days. Colony‐forming units (CFUs) were determined and isolates subcultured on the respective media to obtain pure cultures. For certain hard‐culturable anaerobes such as *Tannerella forsythia* and *Alloprevotella tannerae*, a helper strain (*Propionibacterium acnes* or *Fusobacterium nucleatum*) was applied to the edge of the plate without mixing the strains. Identification of the isolates was performed with classic morphological assessment as well as classical biochemical methods (Gram, catalase, oxidase, and indole reaction), MALDI‐TOF mass spectrometry (Matrix Assisted Laser Desorption Ionization Time of Flight Mass Spectrometry) (Bruker, Billerica/Massachusetts, USA), and 16S rDNA PCR, agarose gel electrophoresis, and, thereafter, Sanger sequencing (GATC Biotech, Konstanz, Germany). Isolates were further classified into groups based on morphological characteristics (cocci, rods), growth conditions (aerobic, anaerobic), and Gram‐staining properties (Gram‐positive, Gram‐negative) (Appendix 1). The whole workflow is illustrated in Figure 1.

### 2.3 Antimicrobial Susceptibility Testing

Antimicrobial susceptibility of all isolated bacteria was verified by disk diffusion (modified Kirby‐Bauer) and, when required, E‐tests for MIC determination. Isolates with reduced susceptibility were further examined for β‐lactamase production using the chromogenic cefinase test (nitrocefin, Becton Dickinson). Disk diffusion test was performed based on EUCAST guidelines (ampicillin 10 µg, tetracycline 30 µg, ciprofloxacin 5 µg). E‐tests were performed for isolates with borderline inhibition zones (≤ 24 mm tetracycline, ≤ 20 mm ciprofloxacin, ≤ 22 mm ampicillin) under aerobic and anaerobic conditions. The incubation time was 18–48 h, but was extended up to 96 h for slow‐growing anaerobes. Bacterial suspensions were prepared in 0.9% NaCl to a turbidity equivalent to McFarland 0.5 (for aerobes) or adjusted visually for anaerobes. If the inoculation load was too low, the test was repeated with a McFarland turbidity of 0.7–1.

The resistance breakpoints used were for tetracycline, a minimum inhibitory concentration (MIC) ≥ 4 mg/L resistant and ≤ 1 mg/L susceptible, for ciprofloxacin, a MIC ≥ 1 mg/l resistant and ≤ 0.25 mg/L susceptible, whereas for ampicillin, species‐ and group‐specific criteria were applied (see Table 1). Intermediate resistance was defined for MIC values between susceptible and resistant thresholds.

**Table 1:** Resistance breakpoints for tetracycline, ciprofloxacin, and ampcillin. Intermediate resistance was defined for MIC values between susceptible and resistant thresholds.

Antibiotic	Bacterial group	Resistant (MIC, mg/L)	Susceptible (MIC, mg/L)
Tetracycline		≥ 4	≤ 1
Ciprofloxacin		≥ 1	≤ 0.25
Ampicillin	Streptococci	≥ 4	≤ 0.5
	Facultative anaerobic Gram‐positive bacteria	≥ 16	≤ 4
	Facultative anaerobic Gram‐negative bacteria	≥ 4	≤ 0.5
	Not further assigned	≥ 16	≤ 2

### 2.4 Statistical Analysis

Statistical analysis was performed using STATA 14.2 (StataCorp LP, College Station, TX, USA). The level of statistical significance was set at *ɑ* = 0.05. Different species were grouped for statistical evaluation. For the analysis of the log_10_‐transformed CPU of the bacteria concentrations mixed linear models were applied to compare the media types as well as the patient groups (healthy‐periodontitis). In case of pairwise comparisons the method of Bonferroni was used to correct for multiple testing. For antibiotic resistance analysis, the Wilcoxon signed‐rank test was applied within the healthy and periodontitis groups to assess differences in values with and without antibiotic treatment. The Wilcoxon rank‐sum test was used to compare results between periodontitis patients and healthy subjects for each cultivation group (defined in Appendix 1). The Friedman test was applied to compare cultivation methods within both groups regarding MIC values. The t‐test was used to compare the two groups (healthy subjects and periodontitis patients) within each cultivation method.

## 3 Results

Samples were taken from 12 patients (healthy group: three males, three females, periodontitis group: three males, three females) with a median (1st; 3rd quartile) age of 27,5 (27; 41) in the healthy group and 65,5 (61; 73) in the periodontitis group. In total, 495 bacterial strains were cultivated (236 from healthy and 259 from periodontitis samples), representing 106 distinct bacterial species. Of these, 54 species (50.9%) were aerobic/facultative anaerobic and 52 (49.1%) obligate anaerobes.

### 3.1 Impact of Selective and Son‐Selective Media on Bacterial Growth

Significant differences in growth were observed across the three media types for almost all bacterial groups, except *S. salivarius* and the Gemella–Granulicatella–Abiotrophia (GGA) group (Appendix 1). Antibiotic‐containing media generally yielded fewer bacteria than non‐selective agar, except for *S. salivarius*, GGA, and *Leptotrichia* spp. (*p* ≤ 0.022). Overall diversity was highest on non‐selective media (*p* = 0.001) and higher on ciprofloxacin than tetracycline agar (*p* = 0.001). When comparing tetracycline‐ and ciprofloxacin‐containing media, significantly fewer bacteria grew on tetracycline agar across most taxa. In contrast, *S. mitis* grew more frequently on tetracycline agar (*p* = 0.0343), while *Actinomyces‐Schaalia* group and *Leptotrichia* spp. grew more on ciprofloxacin agar (*p* = 0.0036 and *p* = 0.0073). No significant differences in growth were observed between healthy and periodontitis groups.

### 3.2 Antibiotic Resistance

**Figure 2:**
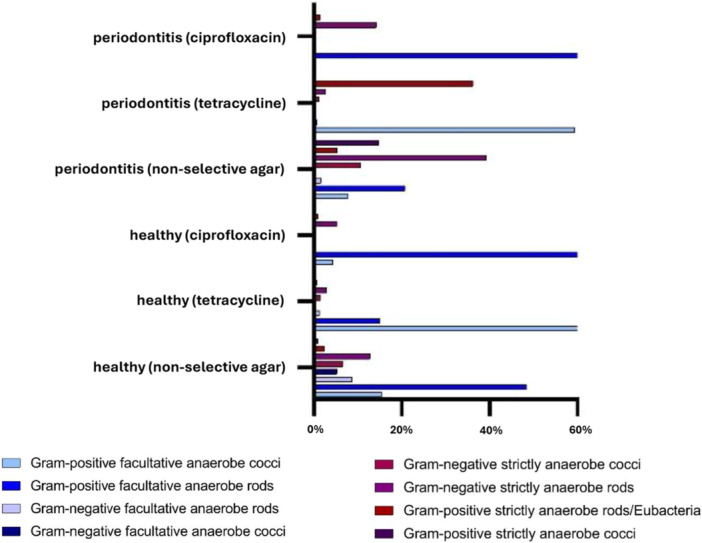
Bacterial distribution of facultative and strictly anaerobic cocci and rods in the healthy and periodontitis groups across different media. On non‐selective, antibiotic‐free agar, Gram‐positive facultative anaerobic rods (48.3%) predominated in the healthy group, whereas Gram‐negative strictly anaerobic rods (39.2%) were dominant in the periodontitis group. On ciprofloxacin‐containing agar, Gram‐positive facultative anaerobic rods were predominant in both groups (healthy: 88.9%, periodontitis: 84.2%), while on tetracycline‐containing agar, Gram‐positive facultative anaerobic cocci (healthy: 78.7%, periodontitis: 59.4%) dominated.

**Figure 3:**
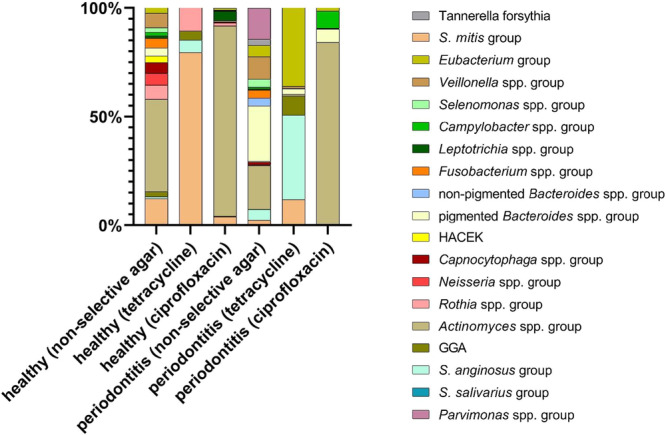
Bacterial distribution on non‐selective, antibiotic‐free agar, tetracycline and ciprofloxacin containing agar in the healthy and periodontitis group. In the healthy group, isolates from 16 different groups were detected on non‐selective media, with *Actinomyces‐Schaalia* group dominating at 42.5%. *S. mitis* (12.3%), *Veillonella* spp. (6.7%), *Rothia* spp. (6.5%), *Neisseria* spp. (5.3%), and *Capnocytophaga* spp. (5.1%) also contributed substantially. All other groups were below 5%. Among periodontitis patients, pigmented *Bacteroides* spp. (29.1%) and *Actinomyces‐Schaalia* group (20.1%) were most frequently isolated on non‐selective agar, followed by *Parvimonas* spp. (14.3%) and *Veillonella* spp. (10.4%). The other groups were ≤ 5.3%. In total, 19 groups were detected in the periodontitis group. The *Streptococcus mitis* group (including *S. oralis, S. mitis, S. cristatus, S. sanguinis, S. parasanguinis*, and *S. gordonii*) was the most frequently tetracycline‐resistant in the healty cohort (healthy 79.6%, periodontitis 11.9%). Other tetracycline‐resistant taxa in the healthy group included *Rothia spp*. (healthy 17.0%), and *S. anginosus* (healthy 5.6%). In periodontitis patients, *S. anginosus* (periodontitis 38.8%) and *Eubacterium* spp. (periodontitis 36.0%) were most common, followed by *S. mitis* group (periodontitis 11.9%). On ciprofloxacin agar, *Actinomyces‐Schaalia* group accounted for the majority of isolates (healthy 87.5%, periodontitis 84.0%).

The number of different cultured species differed significantly between media. On non‐selective agar, a median (IQR) of 18 (17–20) species per sample was detected in healthy and 23.5 (22–26) in periodontitis patients, compared to 5 (4–9) in healthy patients and 6 (5–8) in periodontitis patients on tetracycline agar and 13 (12–16) in healthy and 13.5 (10–15) in periodontitis patients on ciprofloxacin agar. Diversity was significantly higher on non‐selective compared to tetracycline (*p* = 0.001) or ciprofloxacin agar (*p* = 0.001), and also higher on ciprofloxacin than tetracycline agar (*p* = 0.001). Comparison between healthy and periodontitis groups revealed no significant differences in overall microbial growth across media (Figure 2). On non‐selective agar, the most frequent taxa included *Actinomyces‐Schaalia* group (31.9%), pigmented *Bacteroides spp*. (13.9%), *Veillonella spp*. (8.4%), *Streptococcus mitis* group (7.6%), and *Parvimonas spp*. (6.8%). In healthy individuals, *Actinomyces‐Schaalia* group (42.5%) and *S. mitis* (12.3%) predominated. *Veillonella* spp. (6.7%), *Rothia* spp. (6.5%), *Neisseria* spp. (5.3%), and *Capnocytophaga* spp. (5.1%) also contributed substantially. In periodontitis patients, pigmented *Bacteroides* spp. (29.5%) and *Actinomyces‐Schaalia* group (20.1%) were most abundant, followed by *Parvimonas* spp. (14.3%) and *Veillonella* spp. (10.4%) (Figure 3). Generally, in the periodontitis group, aerobes predominated on tetracycline (60.4%) and ciprofloxacin media (85.2%), with unexpectedly low proportions of anaerobes (39.7% and 15.8%, respectively), whereas on non‐selective agar the opposite pattern was observed, with anaerobes clearly dominating (mean 70.0%).

Antibiotic resistance was observed in all subjects. Overall, according to defined MIC values, 75 isolates (15.2%) were resistant to tetracycline, 163 (32.9%) to ciprofloxacin, and 3 (0.6%) to ampicillin. The proportions of resistant isolates were similar between groups (tetracycline: healthy 15.7%, periodontitis 14.7%; ciprofloxacin: healthy 36.0%, periodontitis 30.1%).

**Figure 4:**
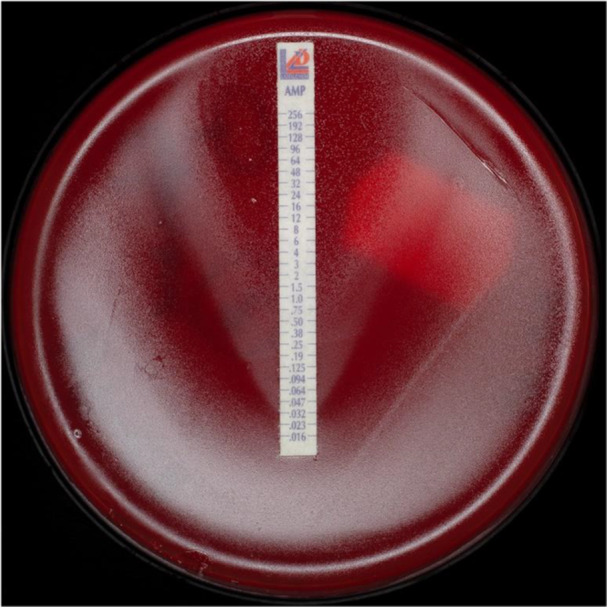
*Fusobacterium nucleatum* isolate showing an unsual growth pattern in an ampicillin E‐test. A noticeable growth inhibition can be seen at < 0.016 mg/L, additional ellipses at 0.32 and 1 mg/L, and some colonies growing along the entire strip up to a MIC > 256 mg/L.

Ampicillin resistance was rare and limited to three isolates: *Haemophilus parainfluenzae* (two isolates from healthy subjects) and *Prevotella oris* (one isolate from a periodontitis patient). Of those three, only one was β‐lactamase‐positive (0.2% of all cultured strains). *Fusobacterium nucleatum* was identified in the oral biofilm samples of all individuals. Three isolates from periodontitis patients exhibited individual colonies reaching the ampicillin disc in the agar diffusion test, despite a general inhibition zone ≥ 30 mm. In subsequent E‐tests, 2 to 4 distinct inhibition ellipses were observed. Single colonies were repeatedly isolated and E‐tests repeated to rule out contamination. Since multiple ellipses persisted, the isolates were classified as generally susceptible to ampicillin. The resistant colonies likely activated resistance mechanisms (Figure 4). The pattern of isolated colonies within the inhibition zone and the presence of multiple inhibition ellipses in the E‐test suggest heteroresistance, where subpopulations exhibit temporarily increased tolerance. Possible underlying mechanisms can include transient β‐lactamase expression or the activation of efflux pumps.

Tetracycline‐ and ciprofloxacin‐resistant isolates were present in all samples. Tetracycline agar generally yielded fewer isolates than ciprofloxacin agar (*p* = 0.001), with significant differences for most groups except *Leptotrichia* spp. and *Eubacterium* spp. The *Streptococcus mitis* group (including *S. oralis, S. mitis, S. cristatus, S. sanguinis, S. parasanguinis*, and *S. gordonii*) was the most frequently tetracycline‐resistant in both cohorts (healthy 43.2%, periodontitis 29.0%). Other tetracycline‐resistant taxa included *Veillonella* spp. (healthy 10.8%, periodontitis 13.2%), *Rothia* spp. (healthy 8.1%, periodontitis 0%), and *Eubacterium* spp. (healthy 8.1%, periodontitis 10.5%). In healthy subjects, *S. mitis* predominated on tetracycline agar (healthy 79.6%, periodontitis 11.9%), while in periodontitis patients, *S. anginosus* (healthy 5.6%, periodontitis 38.8%) and *Eubacterium* spp. (healthy 0.8%, periodontitis 36.0%) were most common. On ciprofloxacin agar, *Actinomyces‐Schaalia* group accounted for 86.5% of isolates (healthy 87.5%, periodontitis 84.0%) (Figure 3). Thus, ciprofloxacin resistance was dominated by *Actinomyces‐Schaalia* group (healthy 27.1%, periodontitis 37.2%), with additional resistant strains among *Rothia* spp. (healthy 9.4%, periodontitis 10.2%), *Leptotrichia* spp. (healthy 15.3%, periodontitis 10.3%), and *Eubacterium* spp. (healthy 14.1%, periodontitis 11.5%). These patterns were largely consistent across healthy and periodontitis subjects, suggesting that differences in resistance profiles reflect intrinsic susceptibility of specific taxa rather than clinical status.

## 4 Discussion

The primary aim of this study was to investigate the phenotypic expression and distribution of antibiotic resistance in culturable oral bacteria in both healthy individuals and those with a history of periodontitis, using antibiotic‐containing agar plates. This approach has not previously been used to examine the antibiotic resistance of oral bacteria. The escalating prevalence of antibiotic‐resistant bacteria, including those in the oral cavity, increasingly complicates clinical treatment outcomes. Oral bacteria are implicated not only in local dental and periodontal diseases but, upon gaining access to the circulation, can cause life‐threatening systemic infections (Kumar 2013). Hence, understanding resistance patterns in the oral cavity is therefore important for both oral and public health. In this study, resistance against tetracycline, ciprofloxacin, and ampicillin was investigated. It is important to note that only 50–65% of known oral species can be cultured (Paster et al. 2006; Aas et al. 2005; Dewhirst et al. 2010; Chen et al. 2010). Therefore, the observed spectrum of this study likely underrepresents the total diversity and repertoire of oral phenotypic resistances.

So far, antibiotic resistance for the investigated antibiotics in the oral cavity has primarily been studied using either previously cultured bacteria or next‐generation sequencing methods such as shotgun metagenomics. While the use of agar plates containing antibiotics is a common approach in many microbiology laboratories, the specific way we applied this method to the antibiotics studied here has, to our knowledge, not yet been reported. As demonstrated by the reduced number of colony‐forming units on selective agar plates containing ciprofloxacin or tetracycline, this technique offers the advantage of preselecting phenotypically resistant oral bacteria, which can then be characterized using more precise, clinically accepted tests, such as disk diffusion (modified Kirby‐Bauer) and E‐tests. Though not all the selected bacteria were revealed to be resistant to ciprofloxacin and tetracycline, the technique used pre‐enriched potentially resistant bacteria.

Although microbial composition differed between groups, no significant differences in overall microbial diversity or resistance prevalence were detected. Periodontitis patients showed a shift toward anaerobic Gram‐negative species such as *Porphyromonas gingivalis* and *Tannerella forsythia* (Walters and Lai 2015; Slots and Ting 1999), whereas healthy individuals were dominated by early colonizers such as *Actinomyces‐Schaalia* group and *Streptococcus mitis (*Li et al. 2004; Kolenbrander et al. 2010). *Parvimonas micra*, a periodontitis‐associated microorganism (Walters and Lai 2015; Cieplik et al. 2019), was found to be significantly more prevalent in patients with periodontitis than in healthy subjects. However, given the limited sample size (n = 12), the relatively small subgroup sizes, and the age differences between the groups, these findings should be interpreted with caution. In particular, the possibility that participant selection may have influenced the observed microbial patterns cannot be excluded, and the lack of comprehensive clinical characterization further constrains confirmatory conclusions.

Generally, in our cohort phenotypic resistance was common. Tetracycline resistance (15.2%) was primarily driven by the *Streptococcus mitis* group (43.2% healthy, 29.0% periodontitis), which could reflect acquired tet(M) mechanisms common in oral streptococci and enabling aerobic predominance under selective pressure (Poutanen et al. 1999). Ciprofloxacin resistance (32.9%), the highest observed, was dominated by *Actinomyces‐Schaalia* group (86.5% of isolates), consistent with earlier results and may be the result of quinolone tolerance e.g. via gyrA variations in actinomycetes (Smith et al. 2005; Cattoir et al. 2010), thus explaining their enrichment on selective media. Ampicillin resistance remained rare (0.6%), limited to sporadic isolates like *Haemophilus parainfluenzae* and *Prevotella oris*, underscoring β‐lactam efficacy against most oral anaerobes. The similar resistance rates in healthy and diseased individuals align with earlier reports of frequently present resistant phenotypes in healthy populations (Anderson et al. 2023), although the presence of resistance genes investigated with PCR was commonly higher (Villedieu et al. 2003). These findings highlight discrepancies between molecular and phenotypic properties, as resistance genes may remain transcriptionally inactive under tested conditions. Thus, some molecular studies showed higher resistance gene frequencies in periodontitis patients (Kim et al. 2011). Interestingly, Arredondo et al. reported a higher proportion of tetracycline‐resistant bacteria in periodontitis patients besides high prevalence of tetracycline‐resistance genes in both groups as shown by further studies (Arredondo et al. 2021; Dave and Tattar 2025). Also Gaetti‐Jardim et al. reported increasing resistance rates with worsening periodontal disease especially in Gram‐negative intestinal rods, with the highest rates observed in edentulous patients (Gaetti‐Jardim et al. 2010). In contrast, our study mainly isolated bacteria from the *Streptococcus* group, *Gemella* spp., *Granulicatella* spp., *Actinomyces‐Schaalia* group, *Neisseria* spp., *Rothia* spp., *Capnocytophaga* spp. and HACEK (Haemophilus‐Aggregatibacter‐Cardiobacterium‐Eikenella‐Kingella‐group). *S. mitis* (28.9%), *Gemella‐Granulicatella‐Abiotrophia* (15.8%) and *Veillonella* spp. (15.8%) exhibited the highest levels of resistance in the periodontitis group. Ciprofloxacin resistance was particularly prevalent, especially in *Actinomyces‐Schaalia* group, consistent with previous findings (Wade 1989; Zaheer et al. 2024). Furthermore, our study expands the known spectrum of ciprofloxacin‐resistant taxa to include *Prevotella* spp. and *Leptotrichia* spp. as well as various Gram‐positive anaerobic rods, underscoring the diversity of the oral resistome (Ehrmann et al. 2016). Thurnheer et al. have already described resistance to ciprofloxacin in *Leptotrichia buccalis* (Thurnheer et al. 2023). Contrary to other studies (Rams et al. 2014; Süzük et al. 2016; Roberts and Kreth 2014), in this study no ciprofloxacin‐resistant *Streptococcus anginosus* could be isolated. Prevalence of tetracycline‐resistance was consistent with previous findings (Lancaster et al. 2005; Zaheer et al. 2024; Rams et al. 2014; Süzük et al. 2016; Roberts and Kreth 2014). Interestingly, in the periodontitis group, aerobes predominated on tetracycline‐ (60.4%) and ciprofloxacin‐containing media (85.2%), contrasting anaerobe dominance (70.0%) on non‐selective agar. This suggests greater intrinsic tolerance of facultative anaerobic taxa like the *Streptococcus mitis* group and *Actinomyces‐Schaalia* group—potentially reflecting natural resistance and biofilm‐driven microenvironmental shifts rather than disease‐specific selection. These findings align with prior observations of *Streptococcus* spp. enrichment under antibiotic pressure in subgingival niches (Zhang et al. 2025).

Surprisingly, ampicillin resistance was rare (0.6%), diverging from earlier studies reporting much higher rates (Gaetti‐Jardim et al. 2010; Ramos et al. 2009). Interestingly, only two of the three ampicillin‐resistant isolates identified (*Haemophilus parainfluenzae, Prevotella oris*) was β‐lactamase positive (0.2% of all isolates). This is in stark contrast to studies reporting up to 82% β‐lactamase‐positive isolates in periodontitis patients (Rams et al. 2013; Ehrmann et al. 2014).

However, studies comparing resistance rates are hampered by differences in inclusion criteria, microbial identification methods (Herrera et al. 2002), and interpretations of resistance thresholds (Veloo et al. 2012). Geographic variations in resistance patterns have also been described (Van Winkelhoff et al. 2005), likely linked to differences in antibiotic consumption and patient compliance (Veloo et al. 2012). Cross‐study comparisons are further limited by the lack of standardized breakpoints and clinical criteria for many oral species—a situation that continues to hinder surveillance and the development of universal recommendations (Veloo et al. 2012).

Our results suggest that antibiotic resistance in the oral microbiota is not solely linked to prior antibiotic use but may also reflect environmental exposure, horizontal gene transfer, or community‐level transmission and question the assumption that oral antibiotic resistance is primarily driven by disease‐associated biofilm disruption, higher inflammation, or local antibiotic therapy. This hypothesis as also supported by Lancaster et al. who showed a high prevalence of tetracycline‐resistance in children without a history of tetracycline exposure (Lancaster et al. 2005). By focusing on phenotypic resistance within the entire culturable flora, our study provides exploratory insights into the oral resistome in health and disease. Of course, the exploratory nature of the study and the small cohort size limit the generalizability of the results. Nevertheless, our findings complement gene‐based approaches (Reynolds et al. 2016; Ehrmann et al. 2017; Roberts and Kreth 2014; Hannan et al. 2010; Mullany et al. 2012; Nguyen et al. 2014), which often identify resistance determinants not manifested phenotypically under laboratory conditions, and underscore the need for larger, well‐characterized clinical datasets to substantiate these observations. Generally, countries with higher consumption of antibiotics generally showed higher numbers of antibiotic resistance genes (Brooks et al. 2022b). Therefore, despite the divergence between genotype and phenotype, antibiotics should be administered in strict accordance with guidelines (Sanz et al. 2020).

## 5 Conclusion

This study confirms the high prevalence of antibiotic resistance in the oral microbiome, even among healthy individuals, by using a new approach of selective antibiotic‐containing agar plates. Pronounced phenotypic resistance to tetracycline and ciprofloxacin was revealed. Comparable resistance rates in health and periodontitis highlight the resilience of resistance within the oral ecosystem. The oral cavity should therefore be regarded as an integral part of the human resistome, as it may serve as a reservoir of resistance genes that can be transferred to pathogenic bacteria, with potential consequences for public health. Future studies should standardize protocols, include larger cohorts, and combine phenotypic with molecular approaches to improve surveillance and informed stewardship.

## Author Contributions

**Marietta Wolf:** conceptualization, formal analysis, investigation, visualization, writing – original draft. **Jennifer Metz:** data curation, formal analysis, investigation, methodology, visualization. **Annette Wittmer:** data curation, methodology, project administration, supervision, writing – review and editing. **Klaus Pelz:** methodology, writing – review and editing. **Kirstin Vach:** formal analysis, methodology, validation, writing – review and editing. **Christiane von Ohle:** funding acquisition, writing – review and editing. **Diana Wolff:** funding acquisition, writing – review and editing. **Cornelia Frese:** funding acquisition, writing – review and editing. **Fabian Cieplik:** conceptualization, funding acquisition, project administration, supervision, writing – review and editing. **Ali Al‐Ahmad:** conceptualization, data curation, funding acquisition, methodology, project administration, supervision, writing – review and editing.

## Ethics Statement

This study was approved by the Ethics Committee of Albert‐Ludwigs‐Universiy, Freiburg, Germany, with the study number 604/16, the Ethics Committee of Heidelberg Medical Faculty, Heidelberg, Germany, with the study number S‐652/2016, as well as by the Ethics Committee of Tuebingen University Hospital, Tuebingen, Germany, with the study number 863/201BO2.

## Conflicts of Interest

The authors declare no conflicts of interest.

## Data Availability

The data that support the findings of this study are available from the corresponding author upon reasonable request.

## Appendix A Overview of the Different Bacterial Groups

**Table:**

Aerobe	Gram‐positive facultative anaerobe cocci	S. mitis group	Streptococcus oralis
			*Streptococcus mitis*
			*Streptococcus cristatus*
			*Streptococcus sanguinis*
			*Streptococcus parasanguinis*
			*Streptococcus gordonii*
		*S. salivarius* group	*Streptococcus salivarius*
			*Streptococcus vestibularis*
		*S. anginosus* group	*Streptococcus anginosus*
			*Streptococcus intermedius*
			*Streptococcus constellatus*
		*Gemella‐Granulicatella‐Abiotrophia* group (GGA)	*Gemella morbillorum*
			*Gemella haemolysans*
			*Gemella sanguinis*
			*Granulicatella adiacens*
			*Abiotrophia defectiva*
	Gram‐positive facultative anaerobe rods	*Actinomyces‐Schaalia* group^***^	*Actinomyces oris*
			*Actinomyces naeslundii*
			*Actinomyces gerencseriae*
			*Actinomyces israelii*
			*Actinomyces dentalis*
			*Actinomyces graevenitzii*
			*Actinomyces oricola*
			*Actinomyces timonensis* *Actinomyces lingnae* *Actinomyces sp. Actinobaculum sp*.
			*Schaalia odontolytica* *Schaalia meyeri* *Schaalia georgiae*
		*Rothia* spp. group	*Rothia mucilaginosa*
			*Rothia dentocariosa*
			*Rothia aeria*
			*Corynebacterium* spp.
	Gram‐negative facultative anaerobe cocci	*Neisseria* spp. group	*Neisseria macacae/mucosa*
			*Neisseria subflava/flavescens/perflava*
			*Neisseria oralis*
			*Neisseria elongata*
			*Neisseria bacilliformis*
			*Lautropia mirabilis*
	Gram‐negative facultative anaerobe rods	*Capnocytophaga* spp. group	*Capnocytophaga ochracea*
			*Capnocytophaga gingivalis*
			*Capnocytophaga sputigena*
			*Capnocytophaga granulosa*
			*Capnocytophaga leadbetterii*
		HACEK group	*Aggregatibacter segnis*
			*Eikenella corrodens*
			*Kingella potus*
			*Cardiobacterium hominis*
			*Cardiobacterium valvarum*
			*Haemophilus parainfluenzae*
		*Ottowia* spp. group	*Ottowia* sp.
		*Candida* spp. group	*Candida albicans*
Anaerobe	Gram‐negative strictly anaerobe rods	pigmented *Bacteroides* spp. group	*Porphyromonas gingivalis*
			*Prevotella nigrescens*
			*Prevotella histicola*
			*Alloprevotella tannerae*
			*Prevotella melaninogenica*
			*Prevotella denticola*
			*Prevotella loescheii*
			*Prevotella conceptionensis*
			pigmented *Bacteroides*
		non‐pigmented *Bacteroides* spp. group	*Prevotella oris*
			*Prevotella oralis*
			*Prevotella nanceiensis*
			*Prevotella heparinolytica*
			*Tannerella forsythia*
		*Fusobacterium* spp. group	*Fusobacterium nucleatum*
		*Leptotrichia* spp. group	*Leptotrichia buccalis*
			*Leptotrichia hofstadii*
			*Leptotrichia trevisanii*
			*Leptotrichia wadei*
			*Leptotrichia goodfellowii*
			*Leptotrichia* sp.
	Gram‐negative strictly anaerobe curved rods	*Campylobacter* spp. group	*Campylobacter rectus*
			*Campylobacter concisus*
			*Campylobacter gracilis*
			*Campylobacter showae*
		*Selenomonas* spp. group	*Selenomonas noxia*
			*Selenomonas artemidis*
			*Selenomonas infelix*
	Gram‐negative strictly anaerobe cocci	*Veillonella* spp. group	*Veillonella parvula*
			*Veillonella dispar*
			*Veillonella atypica*
			*Veillonella sp*.
			*Dialister pneumosintes*
			*Dialister invisus*
			*Anaeroglobus geminatus*
			*Megasphaera micronuciformis*
	Gram‐positive strictly anaerobe rods	*Eubacterium* group	*Atopobium parvulum*
			*Filifactor alocis*
			*Olsenella uli*
			*Olsenella* sp.
			*Mogibacterium timidum*
			*Eubacterium yurii*
			*Lachnoanaerobaculum orale*
			*Lachnoanaerobaculum saburreum*
			*Lachnoanaerobaculum* sp.
			*Johnsonella ignava*
			*Oribacterium* sp.
			*Slackia exigua*
			*Eggerthia catenaformis*
			*Shuttleworthia satelles*
			*Proprionibacterium acnes*
	Gram‐positive strictly anaerobe cocci	*Parvimonas* spp. group	*Parvimonas micra*

**Actinomyces* spp. are classified here as Gram‐positive facultative anaerobic rods. The authors want to note that within this genus, some species (e.g. *A. israelii*, *S. meyeri*) are described as strictly anaerobic.
